# Procalcitonin has a poor prognosis value in critically ill patients with candidemia

**DOI:** 10.1186/cc10638

**Published:** 2012-03-20

**Authors:** PE Charles, R Bruyère, H Roche, JP Quenot, S Prin, A Pavon, F Dalle

**Affiliations:** 1 University Hospital, Dijon, France

## Introduction

Candidemia is an infrequent but serious infection in the critically ill patients. Although effective antifungal drugs are available, mortality rates remain high so far. Procalcitonin (PCT) repeated measurements have proven useful for assessing the prognosis and the antimicrobial treatment responsiveness in the patients with systemic bacterial infection. Little is known about it in the setting of candidemia. The PCT predictive value regarding the outcome of such patients was therefore addressed.

## Methods

A retrospective single-centre observational study. All patients with ICU-acquired pure candidemia between 2005 and 2011 were included. Baseline characteristics and both clinical and biological follow-up data including PCT measurement were collected. The SOFA score was calculated daily during the first week of antifungal treatment. Survivors at discharge from the ICU were compared to nonsurvivors by univariate followed by a Cox regression analysis.

## Results

Fifty patients were included among whom 28 (56%) died in the ICU. *Candida albicans *was the most common isolated yeast (58%), regardless of the outcome. Nonsurvivors were elder and had a greater SAPS II score value on admission than survivors (55.8 ± 21.7 vs. 42.5 ± 14.9 points, *P *= 0.01). The time elapsed between the ICU admission and the onset of invasive candidiasis was significantly longer in the nonsurvivors than in the survivors (8.3 ± 12.8 vs. 1.2 ± 2.8 days, *P *= 0.01). At the onset of candidemia, the nonsurvivors were more severely ill as assessed through SOFA score calculation (10.4 ± 4.4 vs. 7.8 ± 3.9 points, *P *= 0.04). Antifungal treatment was given within the first 24 hours following the onset of candidemia in 60% of the whole patients and was always appropriate, regardless of the survival. During therapy, the SOFA score remained greater in the nonsurvivors than in the survivors. In contrast, PCT failed to differentiate the survivors from the nonsurvivors the day antifungals were started (8.7 ± 13.1 vs. 4.5 ± 4.1 ng/ml, *P *= 0.21), as well as the following days. The SAPS II, the SOFA score and the time elapsed between ICU admission and candidemia onset were the sole independent predictors of death in our study population.

## Conclusion

The late-onset candidemia are more likely to be associated with death than earlier episodes. Unresolved organ failure as assessed through SOFA score despite effective antifungal treatment was associated with death, while PCT failed to predict the outcome.
